# Physical Activity and Depression Among Young Adults in Islamabad: A Cross-sectional Study

**DOI:** 10.7759/cureus.5791

**Published:** 2019-09-28

**Authors:** Rabia Ali, Nismat Javed, Syed M Shah, Robab Naqvi, Zainab Farrukh, Muhammad Salar K Jadoon, Tehreem Tahir, Saima P Iqbal

**Affiliations:** 1 Miscellaneous, Shifa College of Medicine, Shifa Tameer-E-Millat University, Islamabad, PAK; 2 Internal Medicine, Shifa College of Medicine, Shifa Tameer-E-Millat University, Islamabad, PAK; 3 Family Medicine, Shifa College of Medicine, Shifa Tameer-E-Millat University, Islamabad, PAK; 4 Family Medicine, Shifa International Hospitals, Islamabad, PAK

**Keywords:** depression, physical activity, islamabad, gender, young adults

## Abstract

Objective

To determine the relationship between physical activity and depression between the two genders amongst the young adults of Islamabad.

Methods

We conducted a cross-sectional study of students who were studying in various colleges and universities of Islamabad. Students who were willing to participate in the study and who were studying in the institute for more than six months were included in the study. The data was collected through a self-reporting questionnaire and a self-constructed questionnaire. Cronbach's alpha was used to assess the internal consistency of the self-constructed questionnaire, and it was found to be 0.69. The data obtained were analyzed on IBM's Statistical Package for the Social Sciences (SPSS) version 21 (IBM, Armonk, NY, US).

Results

Out of 298 participants, 113 (38%) were males and 185 (62%) were females. The mean age of the participants was 21.60±1.39 years. One-hundred twenty-six participants were found most likely to suffer from depression. Out of these 126 participants, 42 (33%) were females and 84 (67%) were males. Thirty-nine percent of the participants reported fatigue and inability to attend to their normal routine. Pearson correlation was calculated for the association of depression and age, and it was found to be significant (p-value less than 0.05). The correlation for depression with respect to physical activity was also found to be significant (p-value less than 0.05).

Conclusions

Low levels of physical activity can be a major risk factor for the development of depression and the possible exacerbation of any pre-existing mental disorder. There is a need to combat this problem to decrease the use of pharmaceutical means for curing depression.

## Introduction

Depression is characterized by low energy and mood affecting a person’s behavior, feelings, and self-esteem [1]. It is a mental disorder associated with irritability, fatigue, difficulty in concentration, and change in sleeping habits and appetite. The persistence of this condition can increase the severity of the disorder in adulthood. Mismanaged depression will ultimately pose challenges such as poor academics, drug use and abuse, lack of acceptance in social settings, and suicidal tendencies [2]. According to the World Health Organization (WHO), an 18% increase in the number of cases has been reported from 2005 to 2015 [3]. It is imperative to investigate this alarming situation.

Many studies have reported that different factors like health status and lifestyle play an important role in the generation of depression [4]. In developing countries like Pakistan, students spend less time on physical activity (PA) and more on academic studies. As a result of such a sedentary lifestyle, a very large fraction of students is diagnosed with depressive symptoms [5]. A study reported an 8% decrease in depressive symptoms by engaging in one hour a week of PA [6]. In addition to therapy, patients with depression are encouraged to exercise as a coping technique. Patients have reported better self-esteem and improved mood states after regular PA [7].

However, the prevalence of depressive symptoms varies among adolescents, especially with regards to gender. A higher probability of depression has been reported in females as compared to males [8]. Other studies have found greater suicidal tendencies in boys as compared to girls [9]. Levels of physical activity too show gender disparity. By comparing the differences in physical activity levels between genders and their correlation with depressive symptoms, we can better understand the different needs of both the genders. Thus, more appropriate public health guidelines can be made to tackle the issue of depression in society [3]. These public health guidelines can also focus on creating an environment where non-pharmaceutical therapies are incorporated into the management of mental disorders.

The purpose of this study is to highlight the relationship between physical activity and depression among the two genders.

## Materials and methods

We conducted a cross-sectional study on students who were studying in colleges and universities of Islamabad. The target sample comprised students who had been studying for more than six months in the universities and colleges of Islamabad and belong to the age group of 18 to 25 years. The students who were either absent or not officially enrolled in the institute on the day of the study were not included. The sample size was calculated by the World Health Organization sample size calculator to be 400. Informed consent was taken at the beginning of the study and all information provided from the participants was kept confidential.

The study used two questionnaires. The data was first collected using a self-report questionnaire: Moods and Feelings Questionnaire (MFQ). This questionnaire is available free of cost and all information about the questionnaire had been gathered prior to conducting the study [10]. The questionnaire consisted of 33 statements. Each statement had three possible answers: ‘Not true,’ ‘Sometimes,’ and ‘True.’ Each ‘True’ option scored two points, ‘Sometimes’ scored one point and ‘Not true’ scored zero points. All the points for each participant were then added. Any participant with a score equal to and greater than 20 points was considered to have depression.

This questionnaire was followed by another self-constructed questionnaire for physical activity. There were two types of questions asked in this questionnaire. The first question focused on the participant’s habit of exercising if any. The options for this question were ‘Yes’ and ‘No’. The ‘Yes’ response scored one point. The next question focused on the duration of physical activity. The responses and scoring for this question are shown in Table 1.

**Table 1:** Level of exercise and point scores

Duration of exercise (minutes)	Points
0-14	0
15-29	1
30-44	2
45 and above	3

In the end, all the points were added for each participant as a cumulative physical activity score. Cronbach’s alpha was used to assess the internal consistency of the self-constructed questionnaire, and it was found to be 0.69.

The data obtained were analyzed on IBM's Statistical Package for the Social Sciences (SPSS) version 21 (IBM Corp, Armonk, NY, US). Descriptive statistics were used to analyze and describe the data. Frequencies and percentages were calculated for qualitative variables like gender. Mean and standard deviation (SD) were calculated for quantitative variables like age and depression as well as physical activity scores. Pearson’s correlation was also used to determine significant associations.

## Results

A total of 304 questionnaires were completed and six participants did not meet our inclusion criteria. The number of participants in the study was, therefore, 298. Of these, 62% were females and 38% were males. The mean age of the participants was 21.60±1.39 years. One-hundred twenty-six (126) participants had scores higher or equal to 20 and, therefore, were most likely suffering from depression. Out of these 126 participants, 33% were females and 67% were males. The t-test was used to determine significant differences between the two genders in terms of scores. The results of scores for both depression and physical activity are shown in Table 2.

**Table 2:** Depression and physical activity scores among the two genders

	Males	Females	p-value
Mean score of participants suffering from depression	22.7±10.11	25.1±11.23	0.00
Mean score of participants in terms of physical activity	9.4±2.51	7.2±3.33	0.00

The participants had different responses to some of the statements in the Moods and Feelings Questionnaire, as shown in Table 3. 

**Table 3:** Moods and Feelings Questionnaire (MFQ) responses

Statements	Not true (%)	Sometimes (%)	True (%)
I felt miserable or unhappy	23	63	14
I didn’t enjoy anything at all	49	41	10
I was less hungry than usual	49	37	14
I ate more than usual	38	45	17
I felt so tired I just sat around and did nothing	18	43	39
I was moving and walking more slowly than usual	51	30	19
I was very restless	31	42	27
I felt I was no good anymore	43	36	21
I blamed myself for things that weren’t my fault	48	31	21
It was hard for me to make up my mind	26	40	34
I felt grumpy and cross with other people	29	49	22
I felt like talking less than usual	64	27	9
I was talking more slowly than usual	64	27	9
I cried a lot	52	29	19
I thought there was nothing good for me in the future	55	29	16
I thought that life wasn’t worth living	63	25	12
I thought about death or dying	56	27	17
I thought my family would be better off without me	74	15	11
I thought about killing myself	80	14	6
I didn’t want to see my friends	55	32	13
I found it hard to think properly or concentrate	29	42	29
I thought bad things would happen to me	49	29	22
I hated myself	59	23	18
I felt I was a bad person	48	31	21
I thought I looked ugly	46	32	22
I worried about aches and pains	44	31	25
I felt lonely	34	35	31
I thought nobody really loved me	52	28	20
I didn’t have any fun in any of my activities	50	31	19
I thought I could never be as good as other people	45	31	24
I did everything wrong	62	27	11
I didn’t sleep as well as I usually sleep	44	32	24
I slept more than usual	37	36	27

The Pearson correlation was used to find significant associations between depression and other variables, as presented in Table 4.

**Table 4:** Correlation of depression with age and physical activity

VARIABLE	Pearson correlation	P-value
Age	-0.207	0.000
Physical activity	-0.202	0.000

The Pearson correlation for gender and physical activity was found to be - 0.224, which was significant (p-value less than 0.05).

## Discussion

Mental health is one of the main health issues faced by the world. The WHO has stated depression as one of the leading causes of illness and disability in adolescents [11]. At the same time, insufficient physical activity is one of the leading risk factors for death worldwide in all age groups. Globally, one in four adults are not sufficiently active. The WHO recommends that adults aged 18 to 64 years old do at least 150 minutes of moderate-intensity physical activity throughout the week [12].

PA and depression may have a two-way interaction, meaning that depression may cause low levels of PA and, at the same time, low levels of PA can be a risk factor for depression. Many articles show that regular PA reduces the risk of developing depression. Our study showed age and gender being two such factors; however, another study hypothesized that technological advancements have eased daily activities and indirectly influenced physical activity levels [13]. A cross-sectional study carried out in the elderly population of Karachi, Pakistan, show that 40% of the elderly population suffers from depression, further reinforcing the findings of our study [14].

Many studies have been done to observe the effect of leisure-time PA on depressive symptoms. Studies have reported that higher intensity PA reduces the risk of depressive symptoms regardless of frequency or duration of activity [15]. Many ethnicities were also observed in multiple studies, among which African Americans were found to have the most association of PA with depression [16].

In medical practice, the role of PA in the management of depressive symptoms remains difficult to prove. There is a positive dose-response effect for weekly time spent on exercise in minutes per week in reducing depressive symptoms. However, there is no proven correlation found between depressive symptoms and the total length of PA. Hence, it is possible that both short and long-term PA have the same effect on the quality of life and depressive symptoms. This idea is limited by the fact that daily activities are hindered with reduced mobility during aging [17].

One limitation is that many factors were not assessed in the study such as ethnicity, variation in age groups amongst young adults, body mass index, and levels of exercise in terms of minutes. Our study focused on very few educational institutions and, therefore, a comparison could not be made with other cities of Pakistan.

Multicenter analytical studies should be conducted to assess the level of physical activity that is best suited for coping with depression and to identify other risk factors. Institutes must try to promote such activities by providing incentives, for example, extra credit may be rewarded to students who participate in various fitness regimens.

## Conclusions

Low levels of physical activity can increasingly put young adults at risk for depression. Public health guidelines must be established to ensure that, in addition to pharmacological therapies, non-pharmacological coping mechanisms are implemented in the management plan for patients with depression. Additionally, multicentered studies have to be conducted to further find the level of physical activity that is appropriate for the effective management of depressive disorders.
